# On the Practical Applications of the Magnesium Fluorinated Alkoxyaluminate Electrolyte in Mg Battery Cells

**DOI:** 10.1021/acsami.2c05141

**Published:** 2022-06-01

**Authors:** Tjaša Pavčnik, Matic Lozinšek, Klemen Pirnat, Alen Vizintin, Toshihiko Mandai, Doron Aurbach, Robert Dominko, Jan Bitenc

**Affiliations:** † National Institute of Chemistry, Hajdrihova 19, 1000 Ljubljana, Slovenia; ‡ Faculty of Chemistry and Chemical Technology, University of Ljubljana, Večna pot 113, 1000 Ljubljana, Slovenia; § Department of Inorganic Chemistry and Technology, Jožef Stefan Institute, Jamova cesta 39, 1000 Ljubljana, Slovenia; ∥ Center for Advanced Battery Collaboration, Center for Green Research on Energy and Environmental Materials, National Institute for Materials Science, 1-1 Namiki, Ibaraki 305-0044, Japan; ⊥ Chemistry Department and BINA − BIU Center for Nano-technology and Advanced Materials, Bar-Ilan University, Ramat-Gan 5290002, Israel; # Alistore-European Research Institute, CNRS FR 3104, Hub de l’Energie, Rue Baudelocque, 80039 Amiens, France

**Keywords:** Mg battery cells, Chevrel phase, organic cathode, sulfur, electrolyte compatibility

## Abstract

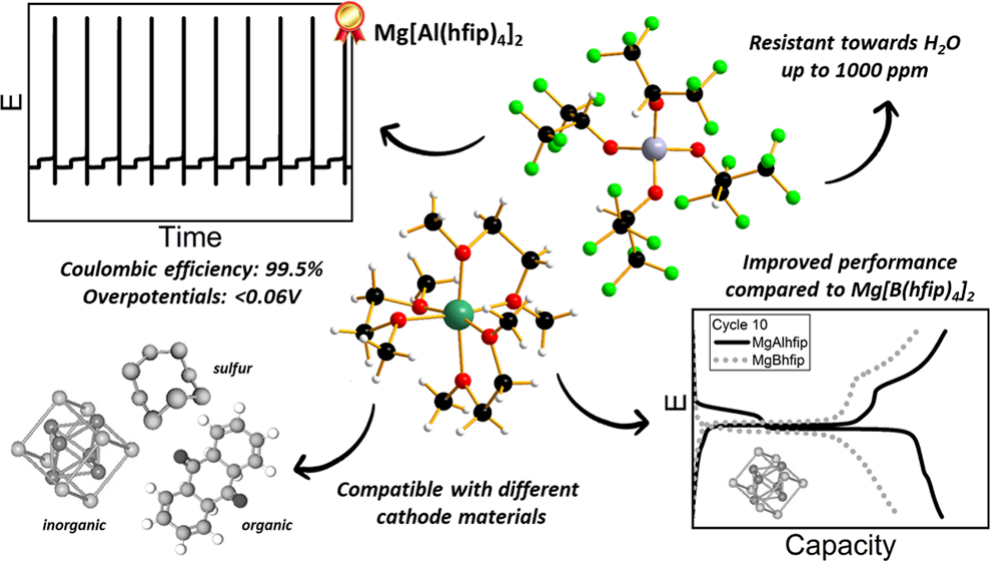

High-performance electrolytes are at the heart of magnesium battery development. Long-term stability along with the low potential difference between plating and stripping processes are needed to consider them for next-generation battery devices. Within this work, we perform an in-depth characterization of the novel Mg[Al(hfip)_4_]_2_ salt in different glyme-based electrolytes. Specific importance is given to the influence of water content and the role of additives in the electrolyte. Mg[Al(hfip)_4_]_2_-based electrolytes exemplify high tolerance to water presence and the beneficial effect of additives under aggravated cycling conditions. Finally, electrolyte compatibility is tested with three different types of Mg cathodes, spanning different types of electrochemical mechanisms (Chevrel phase, organic cathode, sulfur). Benchmarking with an electrolyte containing a state-of-the-art Mg[B(hfip)_4_]_2_ salt exemplifies an improved performance of electrolytes comprising the Mg[Al(hfip)_4_]_2_ salt and establishes Mg[Al(hfip)_4_]_2_ as a new standard salt for the future Mg battery research.

## Introduction

Multivalent metal (Mg, Ca, Al)-based batteries have been long coveted as next-generation battery systems. Mg, Ca, and Al are all rock-forming elements, which means they are geographically evenly distributed and abundant. This eliminates concerns regarding material availability and sustainability that plague the contemporary Li-ion batteries upon rising market demand.^1^ Moreover, multivalent metal anodes offer high gravimetric and volumetric capacities, which enable the design of high-energy-density cells.^2^ Unfortunately, the practical application of rechargeable multivalent metal batteries is lagging due to a small number of available electrolytes in which multivalent metal anodes behave reversibly and the lack of high-energy density cathode materials. Among multivalent metal anode-based batteries, the development of rechargeable Mg battery systems has made the most impressive progress in the last decade with the development of new classes of electrolytes and the application of cathodes that instead of the conventional insertion mechanism operate via conversion or coordination electrochemical reactions.^3^

The importance of high-efficiency metal plating/stripping in connection to rechargeable batteries based on active metal anodes is often underestimated in the literature. In reality, only very high cycling efficiencies above 99% can be considered for practical applications in rechargeable batteries. Even higher efficiencies of more than 99.9% should be targeted for long-term applications above 1000 cycles. For example, if metal plating/stripping efficiency is 99.9%, only 37% of the starting anode capacity is retained after 1000 cycles (Table S1). In practical Mg metal-based battery prototypes, such stringent requirements could be mitigated by the excess of Mg metal anode. Good mechanical properties of the Mg metal mean that Mg foil could serve both as a current collector and anode active material. Already, a relatively thin Mg foil with a thickness of 20 μm has a theoretical areal capacity of 7.7 mAh cm^–2^, by far surpassing the current areal loadings of practical battery electrodes.

The first reversible Mg plating/stripping has been observed in solutions of Grignard reagents almost a century ago.^4^ However, Grignard-based electrolytes are unsuitable as battery electrolytes due to their low oxidative stability and low conductivity. Major progress was achieved through a combination of Grignard reagents with Lewis acids,^5^ which gradually led to the development of the first practical electrolyte for rechargeable Mg battery prototypes and realization of full Mg cells.^6^ Combinations of Grignard reagents and Lewis acids in ethereal solvents enable plating and striping of Mg metal with high Coulombic efficiency (above 99%) and at relatively low overpotentials. Nevertheless, Grignard-based electrolytes contain strong nucleophilic carbon species, which can easily react with electrophilic cathodes like sulfur. Hence, a new class of electrolytes was developed based on a combination of MgCl_2_ with salts like Mg(TFSI)_2_ (TFSI—bis(trifluoromethanesulfonyl)imide), Mg(HMDS)_2_ (HMDS—hexamethyldisilazide), AlCl_3_, etc. in ethereal solvents.^7−9^ The first generation of non-nucleophilic electrolytes contained chloride species, which corrode conventional current collectors (Al, steel, Cu) at elevated potentials. Therefore, a new generation of chloride-free electrolytes was developed based on boron compounds.^10−12^ Among these, the most intensively researched are magnesium fluorinated alkoxy borates that can be prepared through cost-effective transmetalation synthesis.^13^ Further improvement of electrochemical performance of electrolytes based on weakly coordinating anions was achieved by the use of magnesium 1,1,1,3,3,3-hexafluoroisopropoxy (hfip) aluminate salt, Mg[Al(hfip)_4_]_2_, hereinafter denoted MgAlhfip, which displays improved conductivity and higher Mg plating/stripping efficiency with lower overpotentials.^14,15^

Impurities like water, dissolved gasses, and solvent stabilizers among others can have a large detrimental effect on the electrochemical performance of electrolytes for Mg batteries, both from the standpoint of Mg metal plating/stripping efficiency and cells’ overpotential. This can be mitigated by the use of additives with different functionalities, such as impurity scavengers like *n*-Bu_2_Mg^7^ or additives like MgCl_2_ that adsorb to the Mg metal surface, thus reducing its reactivity without a detrimental effect on reversible Mg plating/stripping.^16^ The use of additives can reduce the requirements for solvents and salts quality and/or improve Mg metal plating/stripping efficiency.

In the present work, we synthesized MgAlhfip salt through a modified route.^15^ The obtained salt was characterized using single-crystal X-ray diffraction and combination of IR and NMR spectroscopy. Electrochemical performance of the prepared salt was investigated in different glyme-based electrolytes. In the best performing electrolyte comprising diglyme (G2) as the solvent, Mg metal plating/stripping was studied through different testing protocols and the effect of trace water content and additives was evaluated as well. In the final part, the electrochemical performances of three types of cathodes spanning different types of reaction mechanisms were investigated in MgAlhfip/G2 electrolyte and benchmarked versus electrolyte containing analogous boron salt, Mg[B(hfip)_4_]_2_ (hereinafter denoted MgBhfip).

## Experimental Section

### Electrolyte Synthesis and Characterization

Salt synthesis and electrolyte preparation were performed under an inert atmosphere, in an Ar-filled glovebox with O_2_ and H_2_O levels below 1 ppm. MgAlhfip salt was synthesized following the published procedure,^15^ with modification in the final step of salt isolation. Briefly, 1,1,1,3,3,3-hexafluoroisopropanol (hfipH) (2.5 equiv vs Mg; Apollo Scientific, 99.9%) was dropwise added to 1.0 M *n*-Bu_2_Mg/heptane solution (Sigma-Aldrich). The solvent and residual hfipH were removed under vacuum to obtain the white crystalline powder, Mg(hfip)_2_. The compound was dissolved in 1,2-dimethoxyethane (monoglyme, G1; Sigma-Aldrich, ReagentPlus, ≥99%, inhibitor-free), followed by the addition of 2.0 M Al(CH_3_)_3_/toluene solution (2.02 equiv vs Mg; Sigma-Aldrich). The solution was then cooled to 0 °C, and excess hfipH (3.5 equiv vs Al) was added over a period of 1 h. The reaction mixture was stirred for 24 h at room temperature. Afterward, solvents and residual reactants were removed under vacuum to obtain a concentrated solution, which was slowly added to hexane, in which MgAlhfip salt precipitated. Salt was filtered and dried under vacuum for 24 h at 45 °C. MgBhfip was synthesized according to the published procedure.^13^ Salt was isolated from the reaction mixture in a similar way to MgAlhfip, with precipitation from hexane as described above. HfipH and hexane were dried with 4 Å molecular sieves for 4 days prior to use, whereas G1 underwent an extensive three-step drying procedure consisting of drying with 4 Å molecular sieves for 3 days, one day reflux with Na/K alloy (3:1 wt), and fractional distillation. The amount of water was determined by Karl Fischer titration to be <1 ppm. Other chemicals were used as received.

IR characterization was performed inside the glovebox using an ATR-IR Alpha II (Bruker) equipped with a Ge crystal. Measurements were collected and averaged over 48 scans in the range between 4000 and 600 cm^–1^ with a resolution of 2 cm^–1^. All spectra presented were recorded at room temperature. ^1^H and ^19^F NMR spectra were measured on a Bruker AVANCE NEO 600 MHz NMR spectrometer using DMSO-*d*_6_ solvent.

The sample for single-crystal XRD analysis was grown at −20 °C using the vapor diffusion technique. In total, 50 mg of MgAlhfip salt was placed in a glass vial and dissolved in G1 to make a saturated solution. To promote crystallization, the vial was placed inside a larger vial with approximately 3 mL of *n*-hexane and stored (under a protective Ar atmosphere) in a freezer for 14 days. Single-crystal X-ray diffraction data was measured on a Rigaku OD XtaLAB Synergy-S dual-source microfocus Ag/Cu four-circle diffractometer equipped with an Eiger2 R CdTe 1 M hybrid pixel detector at 100 K. Data collection and processing were performed in *CrysAlis*^*Pro*^ software.^17^*Olex2* (v. 1.5)^18^ was used for structure solution and refinement, employing *SHELXT* and *SHELXL*,^19^ respectively. Molecular graphics were created with the *Diamond*(20) program. Selected crystals from samples covered with a protective layer of perfluorodecalin (abcr, 98%) were attached to MiTeGen MicroLoops with the aid of silicone grease (Bayer) and quickly transferred into the cold nitrogen stream of the diffractometer.

Electrolytes were prepared by adding the appropriate amounts of MgAlhfip or MgBhfip salt and *n*-Bu_2_Mg/MgCl_2_ additives into measuring flasks and diluting them up to the mark to obtain 0.4 M Mg salt/40 mM additive in the G1, diglyme (G2; Acros Organics, 99%, extra pure) or triglyme (G3; Acros Organics, 99%) solvents. Prior to use, solvents underwent an extensive three-step drying procedure described above. The amount of water was determined by Karl Fischer titration to be <1 ppm.

For studying the effect of water on the electrochemical performance of 0.4 M MgAlhfip/G2 electrolyte, G2 solvents with different water contents were used: 0 ppm G2 refers to a dried solvent with an amount of water below 1 ppm; 100 ppm G2 refers to an as-received G2 without any drying and/or purification procedures, in which 125 ppm of water was determined; and 500 and 1000 ppm G2 refer to the as-received G2 solvent with the addition of distilled water, in which 518 and 1037 ppm of water was determined, respectively.

To determine the effect of water presence on the electrolyte composition, NMR spectra of 0.4 M MgAlhfip/G2 electrolytes were measured using 0 and 1000 ppm G2 solvents. Specifically, a few drops of prepared electrolytes were added to the DMSO-*d*_6_ solvent. NMR spectra of electrolytes were measured 24 h after the preparation.

### Material Preparation

Mo_6_S_8_ was prepared by combustion of elemental copper, molybdenum, and sulfur mixtures of 5 g with extra sulfur at a stoichiometry of Cu_2_Mo_6_S_8.5_, which were loaded into Swagelok stainless steel (SS) vessels under an Ar atmosphere. The loaded Swagelok SS vessels were introduced into a hot furnace heated up to 1000 °C for 20 min for a 5 g sample. The ratio between the reactant and total reactor volumes was 1:2. The products were ground by a mortar and pestle to a fine powder of sub-micrometer size particles and were analyzed by XRD as copper Chevrel phases. The copper was extracted by a mild oxidation process in I_2_/acetonitrile solutions. This procedure produced active Mo_6_S_8_ cathode materials.

Polyanthraquinone polymer (PAQ) has been synthesized through cross-coupling polymerization of 1,4-dibromoanthraquinone as described in the literature.^21^ Carbon–sulfur composite was prepared by impregnation of ENSACO 350 G carbon (Imerys Graphite & Carbon) with sulfur to give sulfur carbon composite with 25 wt % sulfur. Briefly, carbon and sulfur were ball-milled for 30 min on a Retsch PM100 at 300 rpm in a mass ratio of 75:25. The mixture was heated in an inert Ar atmosphere in a quartz tube furnace with a heating ramp of 0.2 °C min^–1^ to 155 °C, where it was held for 5 h and cooled afterward to room temperature at a rate of 0.5 °C min^–1^.

### Cathode Preparation

Chevrel phase (Mo_6_S_8_) and organic cathodes were prepared by mixing active material (Mo_6_S_8_ or PAQ) with Printex XE2 carbon black and PTFE binder in a 60:30:10 weight ratio. Sulfur cathodes were prepared by mixing carbon/sulfur composite, multiwalled carbon nanotubes (NTL, M-grade), and PTFE binder in an 80:10:10 weight ratio. All of the components and isopropanol were added into a ball mill jar and homogenized for 30 min on a Retsch PM100 at 300 rpm. Composite matrices were then rolled in between a glass plate and a sheet of baking paper to give self-standing electrodes. Afterward, 12 mm self-standing electrodes were cut, dried, and transferred into the Ar-filled glovebox. Loading of active material was 5.0, 2.0, and 1.2 mg cm^–2^ for Mo_6_S_8_, PAQ, and S active materials, respectively.

### Electrochemical Characterization

Electrochemical testing was performed under galvanostatic mode with a VMP3 potentiostat from Bio-Logic S. A. in two-electrode Swagelok type cells. Cells were assembled with three layers of a glassy fiber separator (GF/A, Whatman, 260 μm) and wetted with approximately 100 μL of electrolyte. Mg foil (0.1 mm, 99.95%, Changsha Rich Nonferrous metals) was polished with P1200 sandpaper inside the glovebox prior to use as an anode. In the first traditional protocol (used for studying the effect of solvents and additives), Mg plating was performed with 1 mA cm^–2^ current density for 60 min and stripping was performed until a cutoff voltage of 2 V. The second employed protocol included macroreversibility measurements,^7^ consisting of plating with 1 mA cm^–2^ for 5 h during which a larger amount of Mg was plated, followed by cycling of only 20% of the plated Mg metal (with 1 mA cm^–2^ current density, 1 h). After 95 cycles in which 20% of the plated Mg was continuously plated/stripped, the remaining Mg metal deposit was stripped until a cutoff voltage of 2 V was reached. The third testing protocol was similar to the first one but included different OCV periods after plating of the Mg metal on Pt working electrodes. To ensure reproducibility of results, all of the galvanostatic Mg stripping/deposition measurements were performed in three parallel cells, and data are presented as the median values. Electrochemical testing of Mg cells with cathodes was done at different C-rates and temperatures. Mo_6_S_8_ cathodes were tested at the C/10 (12.9 mA g^–1^) rate at 50 °C. PAQ polymer cathodes were tested at C/2 (130 mA g^–1^) and sulfur cathodes were tested at C/20 (83.6 mA g^–1^), both at room temperature.

## Results and Discussion

**Figure 1:**
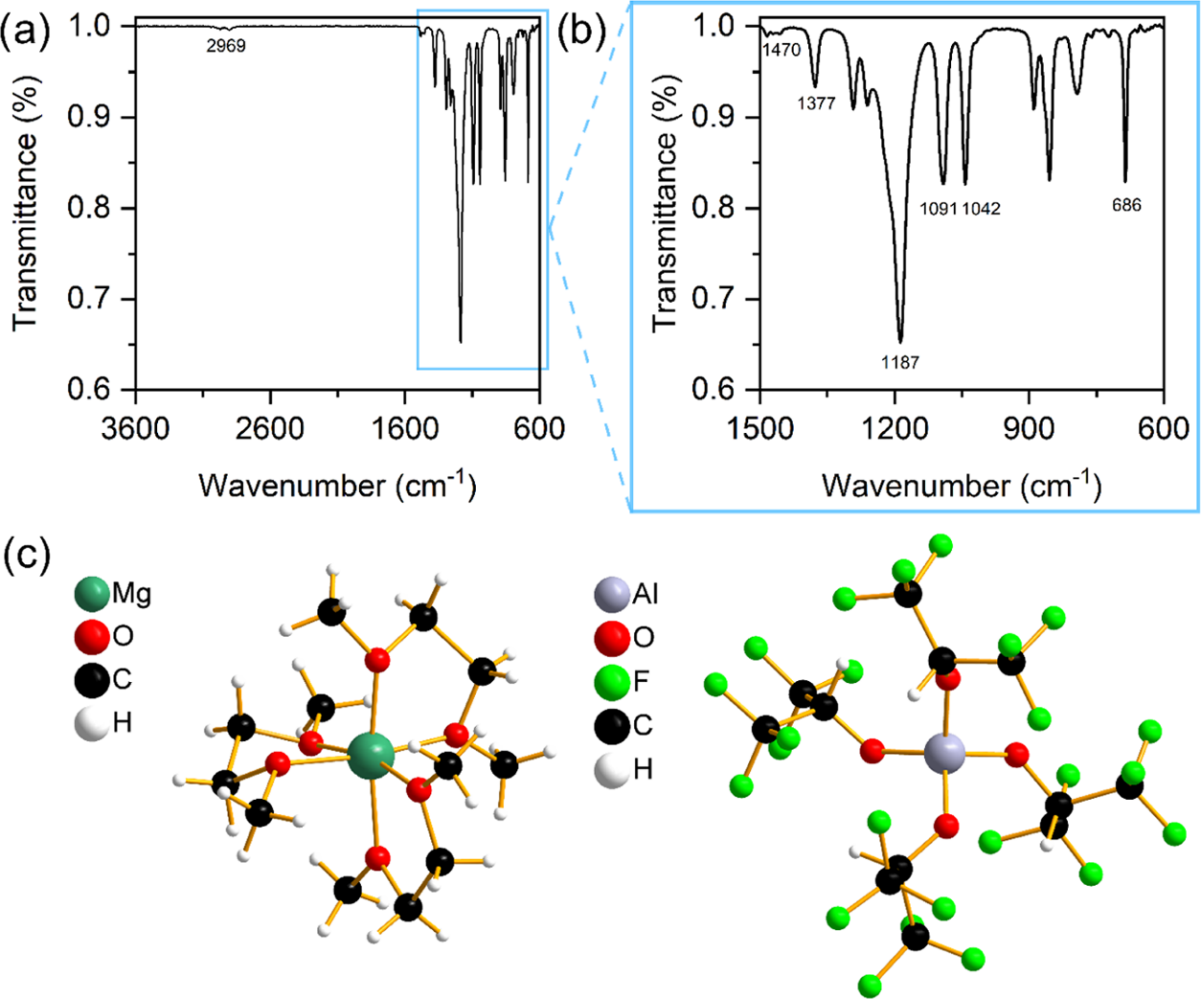
(a) ATR-IR spectrum of MgAlhfip salt with marked characteristic peaks, (b) magnified area below 1500 cm^–1^, and (c) solvated cation [Mg(G1)_3_]^2+^ and [Al(hfip)_4_]^−^ anion of the crystal structure of [Mg(G1)_3_][Al(hfip)_4_]_2_ determined by single-crystal XRD.

A very simplistic calculation of the metal anode capacity retention after long-term cycling at different metal plating/stripping efficiencies shows that Mg plating/stripping above 99.9% should be targeted for realistic Mg metal anode-based battery applications (Table S1). This requirement is far beyond the capabilities of current state-of-the-art electrolytes for Mg batteries, especially if we do not consider nucleophilic and corrosive Grignard-based electrolytes, in which Mg plating/stripping efficiencies above 99% can be achieved quite routinely. A recent discovery of a non-nucleophilic and chloride-free MgAlhfip salt, which exhibits a maximum Mg plating/stripping efficiency above 99%, seems like a promising step toward real application targets.^14,15^ In our work, we synthesized this salt with an added step of salt precipitation from a hexane solution to efficiently remove side products. The as-synthesized salt was characterized by IR spectroscopy (Figure 1a,b), which showed a characteristic broad peak at 1187 cm^–1^ that combines the Al–O–C vibration mode and CF_3_ symmetric and asymmetric stretching modes. Additional peaks for vibration modes of C–CF_3_ groups and deformation mode of the −CF_3_ groups were observed at 1377 and 686 cm^–1^, respectively. A low-intensity peak at 2969 cm^–1^ can be attributed to C–H stretching from the Alhfip^–^ anion, while the broad peak at 1470 cm^–1^ corresponds to the C–H deformation of methyl and methylene groups of the G1 molecules that are coordinated to the Mg^2+^ cation. C–O stretching vibrations from G1 solvent as well as from the aluminum anion are observed at 1091 and 1042 cm^–1^.

^1^H and ^19^F NMR spectra of MgAlhfip salt are in good agreement with the data from the literature (Figures S1 and S2), except the ratio of integrals of anions and G1 protons, which suggests coordination of Mg^2+^ cations with three molecules of G1 solvent and two [Al(hfip)_4_]^−^ anions.^15^ Previously, four G1 molecules were determined through ^1^H NMR, which was also the case in our first measurement. However, upon increasing relaxation times of the NMR experiment, three G1 molecules were determined and it became clear that four G1 determination was an artifact due to the different relaxation times of G1 and [Al(hfip)_4_]^−^ protons (Table S2). Single-crystal XRD measurements on MgAlhfip crystals confirmed the [Mg(G1)_3_][Al(hfip)_4_]_2_ structure (Figure 1**c**). It should be noted that crystals were poorly crystalline with very large unit cells and did not diffract beyond 1 Å. Attempts to obtain higher-resolution data with longer exposure times were unsuccessful due to the crystal decomposition in the X-ray beam of Cu Kα radiation at 100 K. Therefore, the crystal structure could not be satisfactorily refined and only unit cell parameters, space group, and representative molecular structures were reported (Table S3). IR and NMR spectra displaying similar features were obtained during the characterization of the analogous MgBhfip salt (Figures S3–S5), suggesting the [Mg(G1)_3_][B(hfip)_4_]_2_ structure.

**Figure 2:**
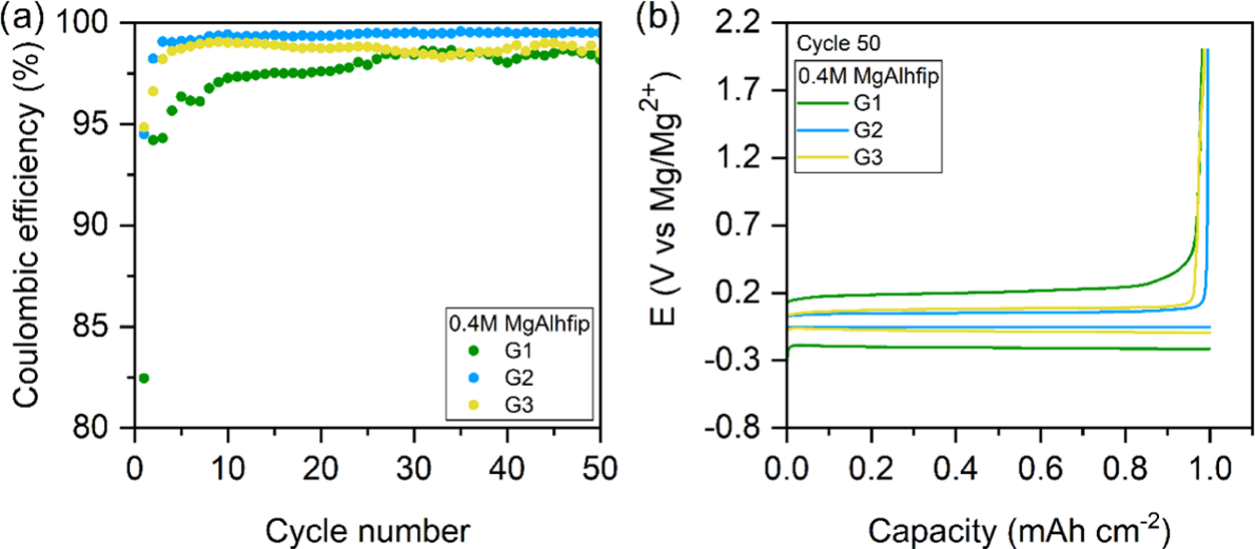
(a) Coulombic efficiency of Mg plating/stripping for 0.4 M MgAlhfip electrolytes in different glyme solvents (G1, green; G2, blue; G3, yellow) and (b) corresponding galvanostatic curves (voltage profiles) for the 50th cycle of Mg plating/stripping measured in long-term cycling experiments with different glyme-base electrolytes. Current density of 1 mA cm^–2^, 1 h Mg plating followed by stripping until an overpotential of 2 V.

The MgAlhfip salt synthesized in this work exhibited good solubility in different glyme solvents, and 0.4 M electrolytes were prepared in monoglyme (G1), diglyme (G2), and triglyme (G3). As-prepared electrolytes were used for Mg plating/stripping tests (Figure 2). All electrolytes displayed a certain activation period related to the electrolytes conditioning, i.e., reaction of remaining impurities.^22^ While the conditioning period in G2 and G3 lasted for only a few cycles, it took 30 cycles with G1-based electrolytes to reach the maximum plating/stripping efficiency. The highest efficiency for Mg plating/stripping processes was achieved in G2-based electrolytes (99.5%), and it remained stable throughout cycling. The efficiency in G3-based electrolytes was slightly lower (99.0%) and starts to fade already after ten cycles. In G1 electrolytes, the maximum efficiency that could be reached was only 98.7%. A similar trend of differences was observed when comparing cells’ overpotentials, G2 and G3-based electrolytes exhibiting 53 and 85 mV, respectively, whereas with G1-based electrolytes a much larger overpotential for Mg plating, up to 206 mV, was measured. Improved electrochemical performance of electrolytes based on G2 and G3 glymes (compared to G1-based electrolytes, as was found in this work) agrees with previous results of similar experiments with electrolytes comprising fluorinated alkoxy borate and aluminate magnesium salts with glyme solvents. The better behavior of G2 and G3 over G1-based electrolytes is associated with improved chelating properties of the longer chains of glyme solvents.^12,13,15^ However, increased viscosity of electrolytes based on glymes with long molecules leads to optimal performance of G2-based electrolytes, which were used for all of the further tests within this work.

**Figure 3:**
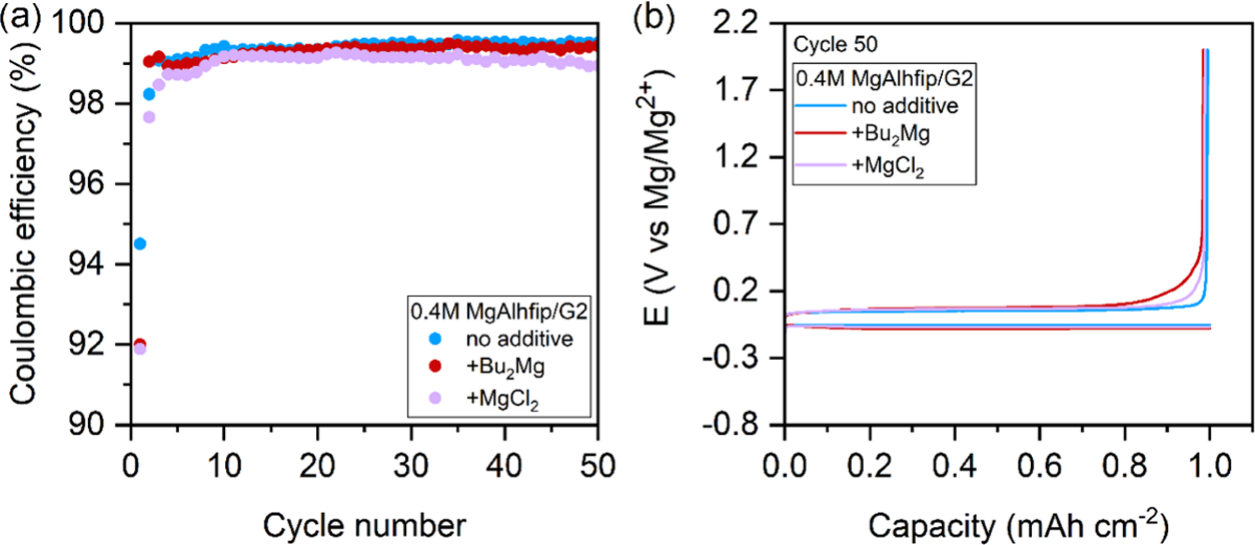
(a) Coulombic efficiency of Mg plating/stripping for 0.4 M MgAlhfip/G2 electrolyte without (blue) and with 40 mM additives (Bu_2_Mg, red and MgCl_2_, violet) and (b) corresponding galvanostatic curves (voltage profiles) for the 50th cycle of Mg plating/stripping measured in long-term cycling experiments. Current density of 1 mA cm^–2^, 1 h Mg plating followed by stripping until an overpotential of 2 V.

The Mg plating/stripping efficiency demonstrated with the MgAlhfip/G2 electrolyte is high but still falls short of the targeted value. Thus, with an aim of further increasing the Coulombic efficiency, two most common electrolyte additives were used, the organometallic protic species scavenging additive (*n*-Bu_2_Mg) and MgCl_2_. The Cl^–^ anions in ethereal Mg salt electrolytes are known to reduce the reactivity of metallic Mg anode surfaces through adsorption to the Mg surface.^16^ Interestingly, no significant improvement was observed by adding them to the MgAlhfip/G2 electrolyte. In turn, electrolytes containing these additives exhibited marginally decreased Coulombic efficiency (Figure 3). Adding *n*-Bu_2_Mg to the electrolyte has an obvious initial positive effect, as it took only one cycle to reach the steady-state Coulombic efficiency above 99% (Figure S6), while with the reference electrolyte longer cycling was required to reach the steady-state Coulombic efficiency of Mg plating/stripping cycles. *n*-Bu_2_Mg is a strong reducing agent that readily reacts with all possible unavoidably present reactive contaminants, thus preventing their reactions on the Mg metal surface, which leads to its passivation.

The overall lack of marked improvement can be explained by the synthesis procedure based on organometallic reagents (Bu_2_Mg and AlMe_3_), which scavenge impurities in the reaction mixture during the salt synthesis, leading to a final salt free from impurities. Moreover, the solvents for the electrolytes were meticulously purified with a multistep drying and distillation procedure ensuring a very low water content, determined by coulometric Karl Fischer titration to be below 1 ppm.

**Figure 4:**
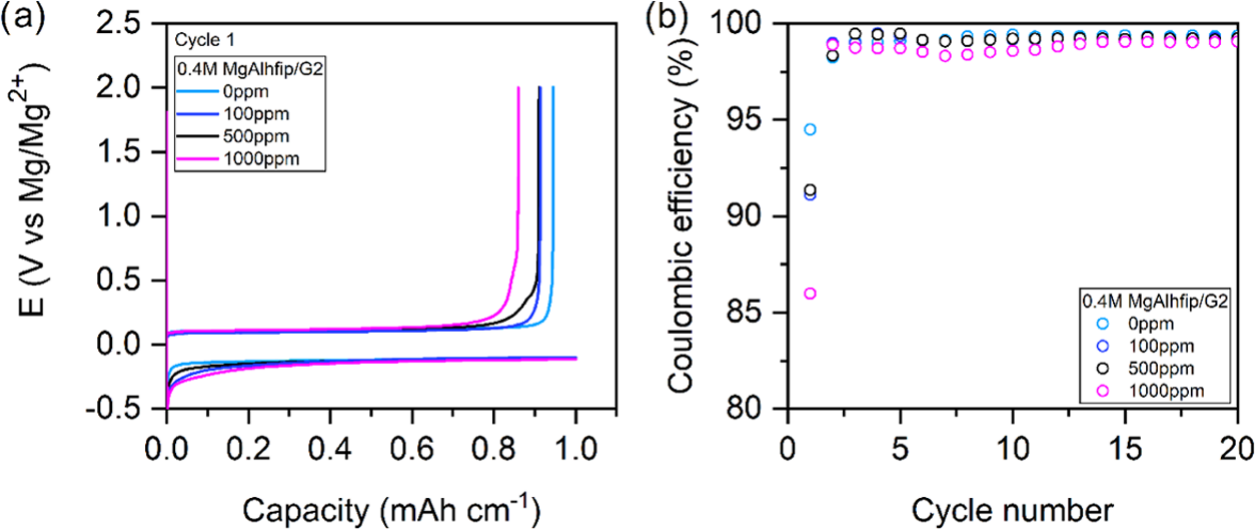
(a) Galvanostatic curves for the first cycle of Mg plating/stripping from 0.4 M MgAlhfip/G2 electrolytes with different water contents. Current density of 1 mA cm^–2^, 1 h Mg plating followed by stripping until an overpotential of 2 V. (b) Coulombic efficiency of Mg plating/stripping.

Rigorous solvent purification and drying are quite time-consuming and require specialized equipment not available in every laboratory. Hence, we decided to test the effect of water content in electrolytes on electrochemical performance. Three additional electrolytes based on G2 solvent with increasing water content (100, 500, and 1000 ppm) were prepared. The water content severely affected the Mg plating/stripping efficiency in the formation cycle: Coulombic efficiency of Mg plating/stripping cycles decreased from 94.5% down to 86.0% with an electrolyte containing 1000 ppm of water. The other two water-contaminated electrolytes performed with Coulombic efficiency between values for the above-mentioned electrolytes (Figure 4). In later cycles, the efficiency of Mg plating/stripping cycles in the water-contaminated electrolytes increased but overall remained lower than the efficiency that could be reached with dry electrolytes. Interestingly, the addition of water did not affect the cells’ overpotentials (Mg plating/stripping processes), which is in sharp contrast with the results of similar experiments with Mg(TFSI)_2_/G1 and MgCl_2_/AlCl_3_/THF electrolytes, where the presence of a few hundred ppm of water increased the difference in the Mg plating/stripping potential to almost 2 and 0.2 V, respectively, and severely decreased the Coulombic efficiency.^16,23^ Anion compatibility with trace water was evaluated through NMR spectroscopy of 0.4 M MgAlhfip/G2 electrolytes with 0 and 1000 ppm of water. The ^1^H NMR spectrum of the water-contaminated electrolyte did not display any new peaks, which excludes the possibility that Alhfip^–^ anions decompose in the presence of trace amounts of water (Figure S7). The relatively high tolerance of the MgAlhfip electrolyte to the presence of water contamination makes it much more transferrable to the practical Mg battery cells.

To better mimic the situation in practical cells, the so-called macroreversibility cycling was developed.^7^ In this procedure, a five times larger amount of Mg metal is plated on the working electrode (WE) and then only one-fifth of the Mg metal is cycled until the final stripping of the Mg metal reaches the cutoff voltage. A downside of this procedure is that only the average Coulombic efficiency of the whole cycling procedure can be obtained. A specific difference in this procedure is that a large amount of Mg metal is plated on the WE in the first cycle. Cycling only 20% of the plated Mg in the successive cycles means that the plating and stripping processes resemble cycling experiments of Mg||Mg symmetrical cells. In these experiments, the large amount of plated Mg metal, which is present on the WE throughout the entire cycling experiments, can amplify the passivation phenomena caused by the side reactions of the plated Mg with components of the electrolytes (Figure S8). A comparison of the average Coulombic efficiency for regular cycling procedures and macroreversibility procedures displays very similar electrochemical performance. The only difference is a slight decrease of the average Coulombic efficiency by 0.2–0.4% (Table 1). This points to a possibility of some Mg metal passivation that could be attributed to the decomposition of Mg^2+^–solvent complexes and some possible salt anions’ decomposition on the surface of the Mg metal anodes.^24^

**Table 1:** Average Coulombic Efficiency of 0.4 M MgAlhfip/G2 Electrolytes without and with Additives^a^

electrolyte	traditional cycling efficiency (%)	macroreversibility efficiency (%)
0.4 M MgAlhfip/G2	99.3	98.9
+40 mM Bu_2_Mg	99.2	98.8
+40 mM MgCl_2_	98.9	98.7

a Comparison of the Coulombic efficiency for the traditional cycling procedure (average value for 100 cycles is reported) and macroreversibility Mg plating/stripping.

To further accelerate possible Mg metal passivation, we performed Mg plating/stripping experiments in various electrolytes with added open-circuit voltage (OCV) periods after Mg plating on Pt WE. This should amplify spontaneous passivation processes due to an extended period of time when Mg metal deposits are in contact with the electrolyte. First, 20 cycles of Mg plating/stripping without OCV pause periods were performed. Afterward, open-circuit voltage pause periods were added after each Mg plating half-cycle (1 or 2 h). Such methodology enables us to emphasize the effect of additives on cycling efficiency. Both additives, *n*-Bu_2_Mg and MgCl_2_, were tested. The improvement in the Coulombic efficiency in these experiments owing to the presence of additives was 1.3% for *n*-Bu_2_Mg and 1.6% for MgCl_2_ in comparison with electrolytes without additives (difference of the average value of ten Mg plating/stripping cycles) for the OCV pause period of 2 h between Mg plating and stripping phases of the cycle (where the differences were the largest, Figure S9). Additionally, there is some difference in the Mg stripping voltage profile. The slope of the curve decreased toward the end of the cycles, as measured with the reference electrolyte without additives in experiments that included OCV pauses. Such behavior could be attributed to the Mg metal deposits’ passivation, to which both *n*-Bu_2_Mg and MgCl_2_ additives display a positive effect, as the slope of curves during cycling remains stable. The best performance of electrolytes containing MgCl_2_ additives hints that the surface coverage of Mg metal anodes by adsorbed chloride anions might be more important than scavenging impurities from the electrolyte by Bu_2_Mg. Overall, these more stringent Mg metal plating/stripping tests exemplify that the quality of these electrolytes and the reversible behavior of Mg anodes in them should and can be further improved for practical applications by the use of functional additives. Furthermore, MgAlhfip-based electrolytes, while already showing exceptional electrochemical performance, can still benefit from the use of functional additives, which opens the possibility of raising the reversibility of Mg anodes in modified electrolytes close to a cycling efficiency of 100%.

**Figure 5:**
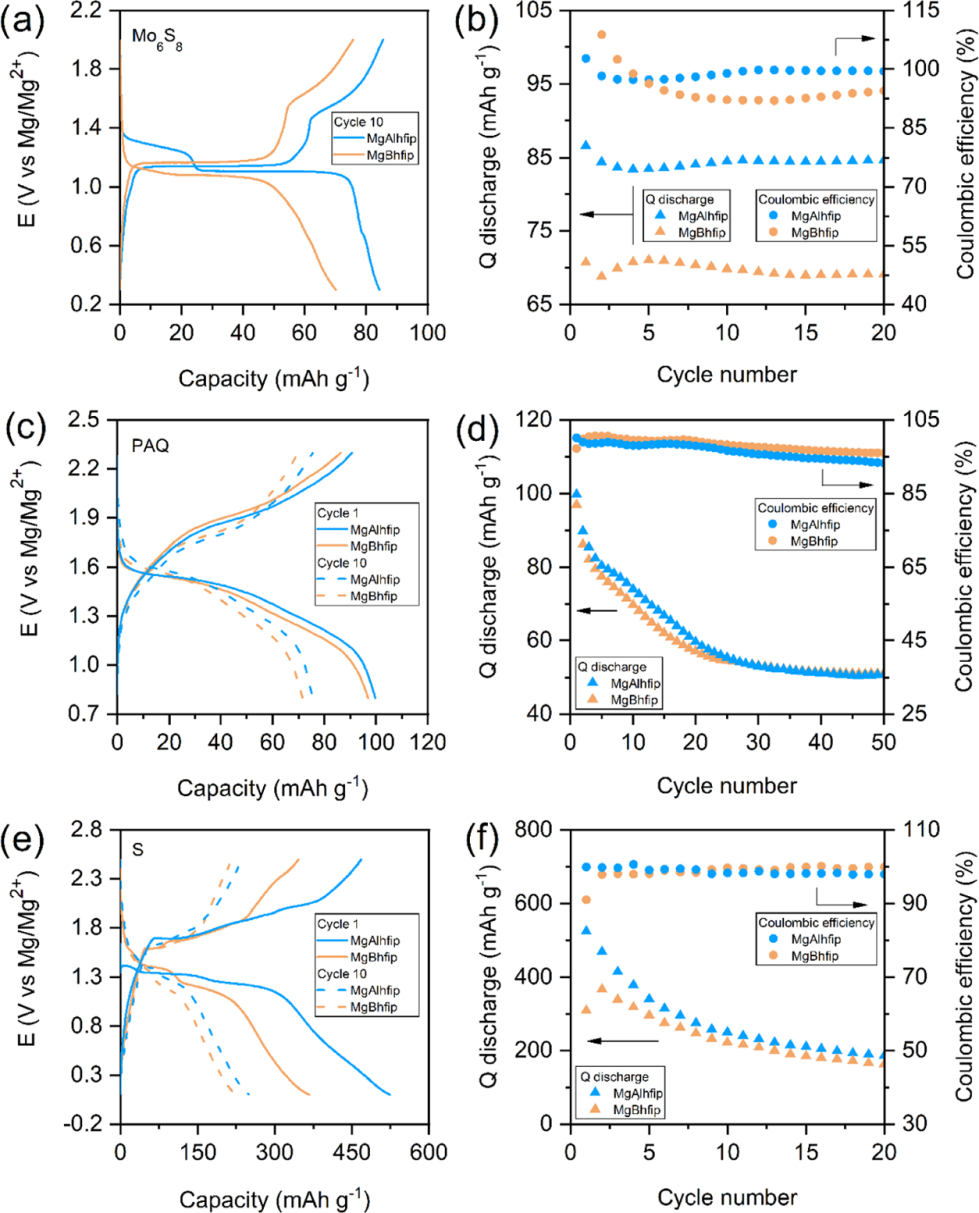
Comparison of electrochemical performance of Mg cells containing 0.4 M MgAlhfip (blue) and MgBhfip (orange) electrolytes in G2 glyme, with different cathodes. Chevrel phase: (a) Discharge/charge voltage profiles in cycle 10 at C/10 in the voltage window from 0.3 to 2.0 V and (b) discharge capacity and Coulombic efficiency. PAQ: (c) Discharge/charge voltage profiles in cycles 1 (solid) and 10 (dashed) at C/2 in the voltage window from 0.8 to 2.3 V and (d) discharge capacity and Coulombic efficiency. Sulfur: (e) Discharge/charge voltage profile in cycles 1 (solid) and 10 (dashed) at C/20 in the voltage window from 0.1 to 2.5 V and (f) discharge capacity and Coulombic efficiency.

In the next step, the compatibility of MgAlhfip electrolytes with different types of cathodes was tested, namely, with Chevrel phase-Mo_6_S_8_, redox-active organic polymer, and sulfur. These three cathode materials undergo different electrochemical reactions with Mg^2+^ ions through different mechanisms (insertion, coordination, and conversion), and thereby, the three of them were chosen for this demonstration (Figure 5). Chevrel phase is a standard insertion material, typically used for benchmarking the performance of different novelties in Mg batteries. The electrochemical performance of MgAlhfip was compared to that of MgBhfip in G2-based electrolytes (Figure 5a,b). Mg||Chevrel phase cells containing the MgAlhfip/G2 electrolyte demonstrate a better capacity utilization and lower overpotentials. After ten cycles, the capacity of these cells stabilizes at around 85 mAh g^–1^, whereas the capacity of similar cells containing the MgBhfip/G2 electrolyte is around 70 mAh g^–1^ (theoretical capacity 122 mAh g^–1^). A peculiar difference is also observed in the shape of the discharge voltage plateau, which is exemplified as a pronounced voltage plateau at 1.25 V, pointing to the reduced trapping of Mg^2+^ ions inside the structure and better utilization of the second site for Mg^2+^ ion insertion inside the Mo_6_S_8_ structure^25^ already at a relatively low temperature of 50 °C. Both experiments also show that the presence of Cl^–^ ions is not needed for a reversible electrochemical performance of Chevrel phase cathodes, while it may be mandatory for reversible Mg^2+^ ions interactions (anodes and cathodes) in Mg(TFSI)_2_/G1 electrolytes.^26^ Difficult Mg^2+^ ion insertion into inorganic hosts like transition metal oxides has prompted researchers to pursue alternative cathode materials.^27^ Among them, organic compounds with redox activity and sulfur have attracted a lot of attention. Due to their relatively soft structure, organic molecules can easily accommodate Mg cations. Anthraquinone-based cathodes typically serve as a model system and display good reversibility.^21,28^ Cathodes comprising polyanthraquinone (PAQ, theoretical capacity of 260 mAh g^–1^) polymer exemplify high capacity utilization in Mg cells in the initial cycles followed by a sharp decrease in the capacity that becomes more gradual in the later cycles. The performance of Mg cells employing PAQ cathodes using either MgAlhfip or MgBhfip electrolytes is very similar, both in terms of discharge capacity and Coulombic efficiency (Figure 5c,d). A clear difference can be observed in terms of cells’ overpotential, where again MgAlhfip exemplifies lower overpotential throughout the cycling experiments. Overall, the reversibility of PAQ electrodes in Mg cells is good. However, PAQ suffers from capacity fading, which could be explained by polymer swelling and partial dissolution of the active mass in cells containing nonconcentrated electrolytes.^21,29^

Sulfur is a promising cathode due to its high theoretical capacity and high natural abundance. Hence, Mg–S batteries have been intensively researched in the last years, specifically after the introduction of the electrolytes that enabled reversible electrochemical cycling of sulfur cathodes.^30−32^ A maximum capacity of 524 mAh g^–1^ (the theoretical capacity is 1672 mAh g^–1^) is achieved in the first cycle upon cycling Mg–S cells containing the MgAlhfip electrolyte (Figure 5e,f). This is followed by a rapid capacity fade in initial cycles, which is quite typical for sulfur cathodes in Mg systems. A comparison between the two electrolytes shows again higher discharge capacity and higher Coulombic efficiency of S cathodes in the MgAlhfip electrolyte. However, similar to the case of Mg cells with the organic cathodes, there is no improvement in long-term capacity retention, exemplifying that the operation mechanism of the sulfur cathodes in Mg cells does not depend on the type of salt in the electrolyte. A comparison of selected galvanostatic curves displays multiple plateaus due to electrochemical conversion of different polysulfide species formed upon sulfur reduction and lower overpotential of cells containing the MgAlhfip electrolyte.

## Conclusions

In this work, the structure of the MgAlhfip salt was determined by single-crystal XRD and complemented by NMR and IR characterization. The performance of the MgAlhfip salt was investigated in different ether type solvents, from an application point of view. Mg plating/stripping behavior in prepared electrolytes was evaluated at realistic conditions by plating and striping 1 mAh cm^–2^ Mg metal at a current density of 1 mA cm^–2^. The electrochemical performance was optimized by examining a variety of different glyme solvents, from which G2 was chosen as an optimal glyme solvent, in which the plating/stripping efficiency of magnesium was the highest, 99.5%. The MgAlhfip/G2 electrolyte exhibited high tolerance to the presence of trace water. Even with the MgAlhfip/G2 electrolyte containing 1000 ppm of water, it was possible to plate and strip magnesium reversibly (at around 85% efficiency in the first cycle and efficiency of more than 98% in later cycles). This high tolerance of MgAlhfip/G2 to water contamination (i.e., no sign of any irreversible side reactions of the electrolyte) should greatly simplify electrolytes’ preparation for practical Mg batteries. Mg plating/stripping cycling performance was evaluated using more representative macrocycling conditions and via cycling experiments that include pause periods at OCV between parts of the half-cycle. Both protocols amplify side reactions and Mg passivation phenomena when they exist. Thereby, such experiments are very suitable to examine the effect of additives that may be capable to mitigate the detrimental passivation phenomena. Indeed, experiments, which included pause periods between cycling, clearly demonstrate the positive effect of two additives, *n*-Bu_2_Mg and MgCl_2_ on the cycling efficiency of Mg plating/stripping processes in the MgAlhfip/G2 electrolyte. Both additives are relevant for the long-term operation of practical Mg cells based on the MgAlhfip/G2 electrolyte. Finally, the performance MgAlhfip/G2 electrolyte was evaluated in Mg cells that contained three different types of cathodes, benchmarked in analogous experiments with a reference MgBhfip/G2 electrolyte. These cathode materials included an inorganic Chevrel phase, redox-active organic cathode polyanthraquinone (PAQ), and sulfur. Cells comprising different cathodes, Mg anodes, and MgAlhfip/G2 electrolytes exemplify reversible electrochemical behavior with reduced overpotential, compared to the reference cells with MgBhfip electrolytes.

## Supporting Information Available

Calculation of metal retention after different numbers of metal plating/stripping cycles; NMR, IR, and XRD characterizations; and additional electrochemical data (PDF)
