# Supplementary material for: On the Practical Applications of the Magnesium Fluorinated Alkoxyaluminate Electrolyte in Mg Battery Cells
Source: ACS Appl Mater Interfaces. 2022 Jun 1;14(23):26766–74. doi: 10.1021/acsami.2c05141 (PMC9204688; doi:10.1021/acsami.2c05141)
Supplement: Supplementary file 1 — am2c05141_si_001.pdf [file am2c05141_si_001.pdf]

Supporting Information

On the practical applications of the magnesium fluorinated alkoxyaluminate electrolyte in Mg battery cells

Tjaša Pavčnik[1,2], Matic Lozinšek[3], Klemen Pirnat[1], Alen Vizintin[1], Toshihiko Mandai[4], Doron Aurbach[5], Robert Dominko[1,2,6], Jan Bitenc[1*]

[1] National Institute of Chemistry, Hajdrihova 19, 1000, Ljubljana, Slovenia

[2] Faculty of Chemistry and Chemical Technology, University of Ljubljana, Večna pot 113, 1000, Ljubljana, Slovenia

[3] Department of Inorganic Chemistry and Technology, Jožef Stefan Institute, Jamova cesta 39, 1000 Ljubljana, Slovenia

[4] Center for Advanced Battery Collaboration, Center for Green Research on Energy and Environmental Materials, National Institute for Materials Science, 1-1 Namiki, Ibaraki 305-0044, Japan

[5] Chemistry department and BINA – BIU center for nano-technology and advanced materials, Bar-Ilan University, Ramat-Gan 5290002, Israel

[6] Alistore-European Research Institute, CNRS FR 3104, Hub de l'Energie, Rue Baudelocque, 80039, Amiens, France

[*]Corresponding author: jan.bitenc@ki.si

Table S1: Calculation of metal retention after different number of metal plating/stripping cycles under conditions of different single cycle metal plating/stripping efficiency. Values of calculated metal retentions below 0.001% are not displayed (/). The calculations are based on the consideration that electrode capacities are evenly matched and all the available metal is cycled in every cycle with constant plating/stripping efficiency throughout the cycling. For capacity retention of practical Mg battery cells also Coulombic efficiency of cathode reaction and exact electrode capacity matching should be considered.

**Single cycle plating/stripping efficiency**	**Retention of metal anode after respective number of cycles**			
	**10**	**100**	**1000**	**10 000**
**90%**	35%	0.0027%	/	/
**95%**	60%	0.59%	/	/
**98%**	82%	13%	/	/
**99%**	90%	37%	0.0043%	/
**99.9%**	99%	90%	37%	0.0045%
**99.95%**	99.5%	95%	61%	0.7%
**99.99%**	99.9%	99.0%	90%	37%

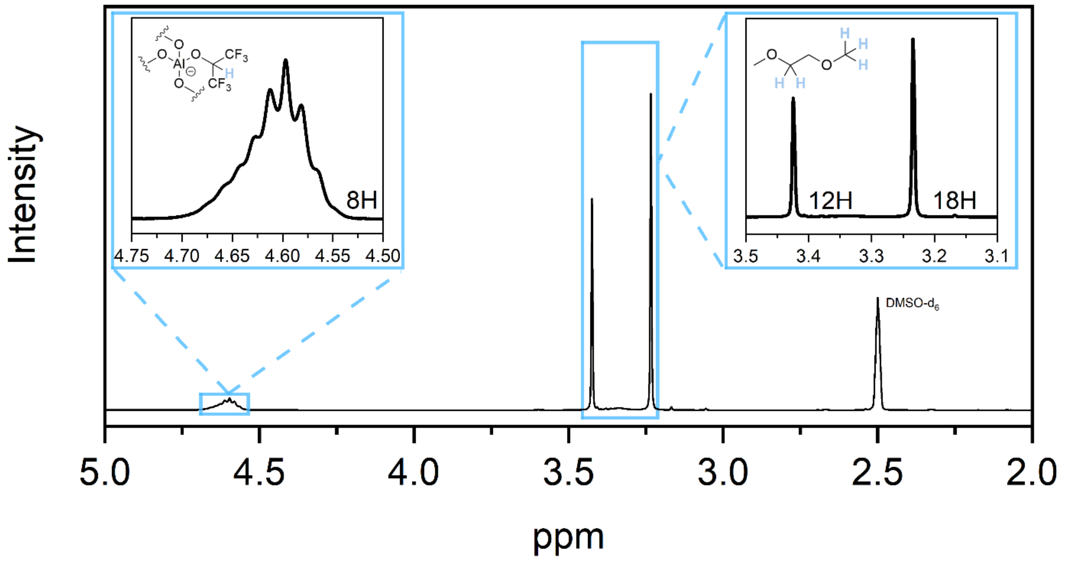

Figure S1: ^{1}H NMR spectrum of $[Mg(G1)_3][Al(hfip)_4]_2$ salt. ^{1}H NMR (600 MHz, DMSO-d_6) δ 4.73–4.51 (m, 8H), 3.42 (s, 12H), 3.23 (s, 18H).

Table S2: Influence of relaxation time on ratio of integrals for protons from $[Al(hfip)_4]_2^-$ anion and from G1 solvent. Increased relaxation time supports the ratio of integrals corresponding to 3 molecules of G1 coordinated to the Mg^{2+} cation. Minor deviations from the expected values are observed due to the overlap with satellite signals.

**Ratio of integrals**	**$[Al(hfip)_4]_2^-$**	**G1**	
	–OC**H**(CF$_3$)$_2$	–C**H**$_2$–	–C**H**$_3$
**expected**	8	12	18
5 s delay	8	15.21	22.91
30 s delay	8	13.53	20.50
120 s delay	8	13.16	19.54

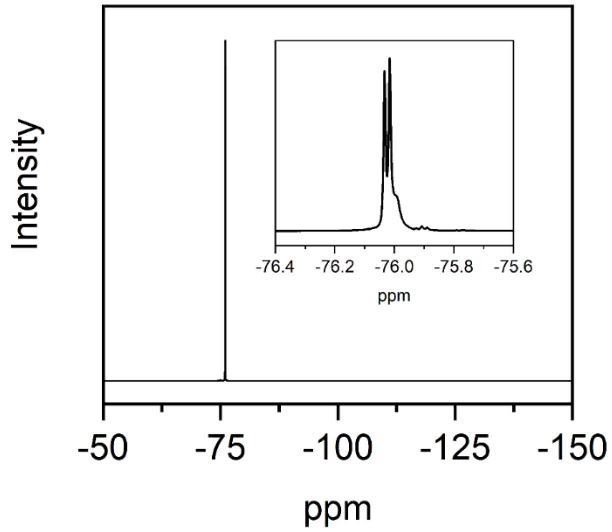

Figure S2: ^{19}F NMR spectrum of $[Mg(G1)_3][Al(hfip)_4]_2$ salt. ^{19}F NMR (565 MHz, DMSO-d_6) δ −76.02 (d, J = 6.5 Hz).

Table S3: Summary of the crystal data of $[Mg(G1)_3][Al(hfip)_4]_2$.

**Compound**	$[Mg(G1)_3][Al(hfip)_4]_2$
Formula	$C_{36}H_{38}Al_2F_{48}MgO_{14}$
F_w	815.65
T [K]	100.0(1)
Crystal system	Monoclinic
Space group	$P2_1/c$
a [Å]	20.4836(7)
b [Å]	50.952(2)
c [Å]	19.7129(6)
α [°]	90
β [°]	114.685(4)
γ [°]	90
V [Å^3]	18694(1)
Z	12
Z'	3
Radiation type	Cu $K\alpha$
λ [Å]	1.54184

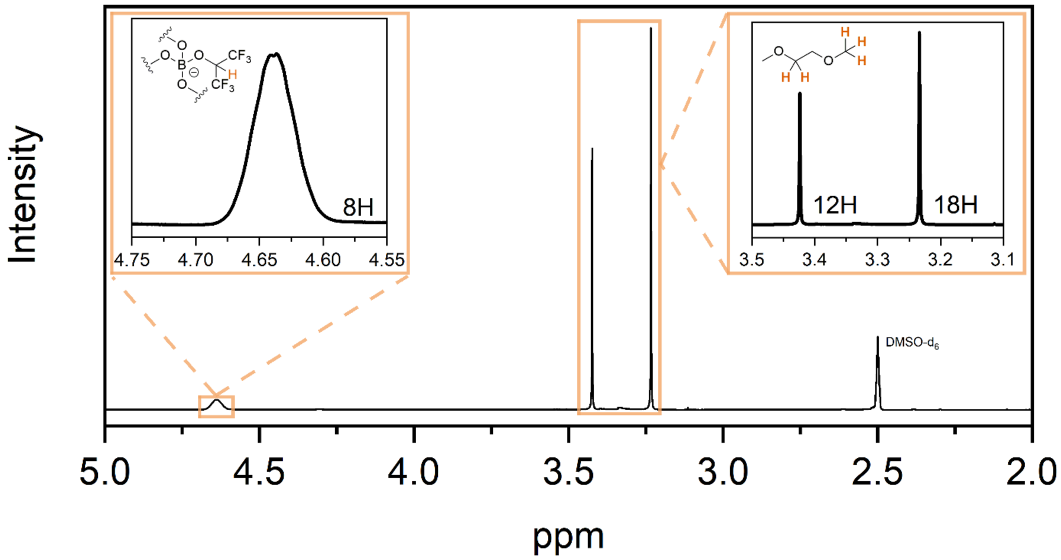

Figure S3: ^{1}H NMR spectrum of $[Mg(G1)_3][B(hfip)_4]_2$ salt. ^{1}H NMR (600 MHz, DMSO-d_6) δ 4.68–4.59 (m, 8H), 3.42 (s, 12H), 3.23 (s, 18H).

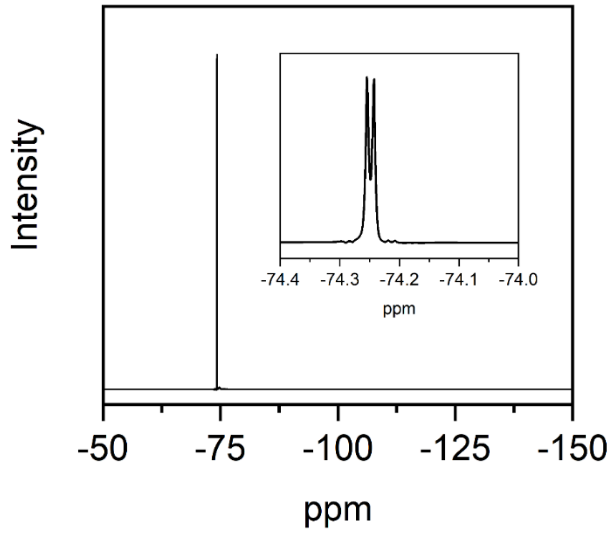

Figure S4: ^{19}F NMR spectrum of $[Mg(G1)_3][B(hfip)_4]_2$ salt. ^{19}F NMR (565 MHz, DMSO-d_6) δ −74.25 (d, *J* = 6.3 Hz).

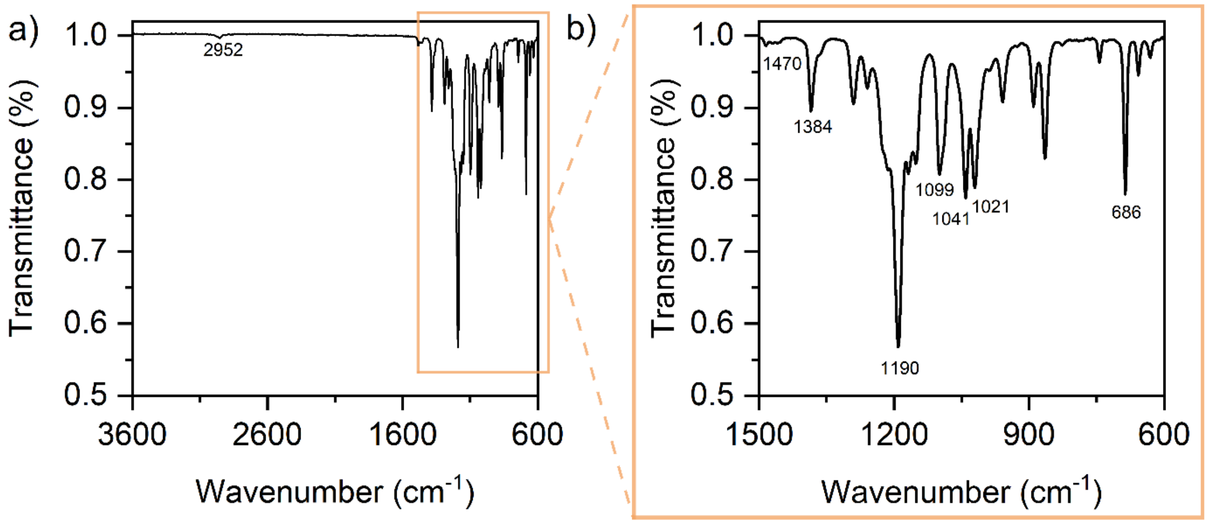

Figure S5: a) IR spectrum of MgBhfip salt with marked characteristic peaks, b) Magnified area below 1500 cm^{-1}.

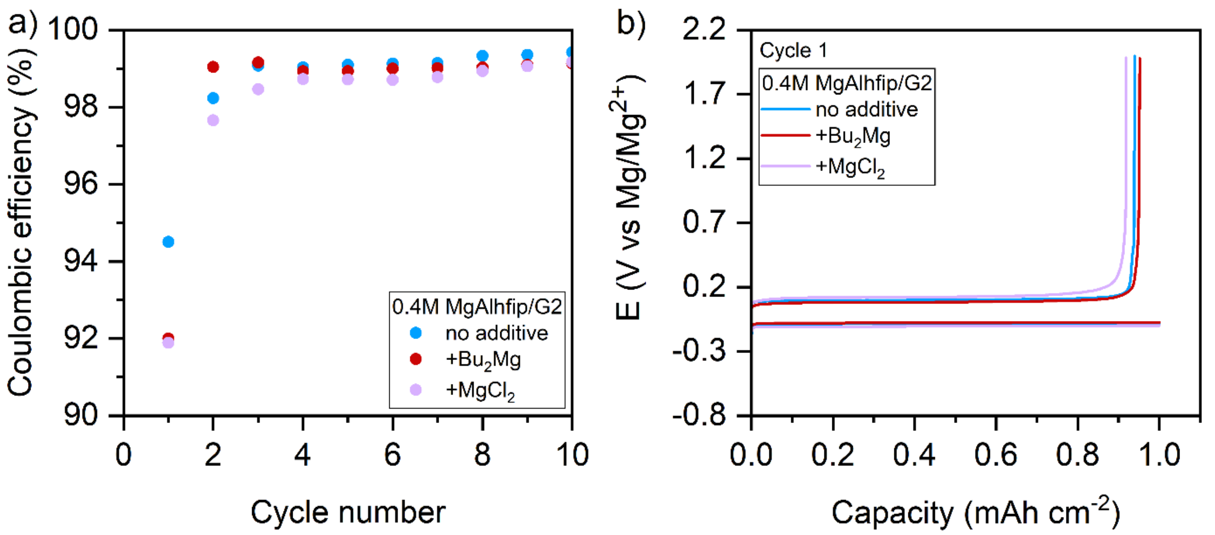

Figure S6: a) Coulombic efficiency of Mg plating/stripping for 0.4 M MgAlhfip/G2 electrolytes without and with 40 mM additives (Bu_2Mg-red and $MgCl_2$-violet) in initial cycles, b) The corresponding galvanostatic curves (voltage profiles) for the 1st cycle of Mg plating/stripping measured in long-term cycling experiments. Current density 1 mA cm^{-2}, 1 h Mg plating followed by stripping until overpotential of 2 V.

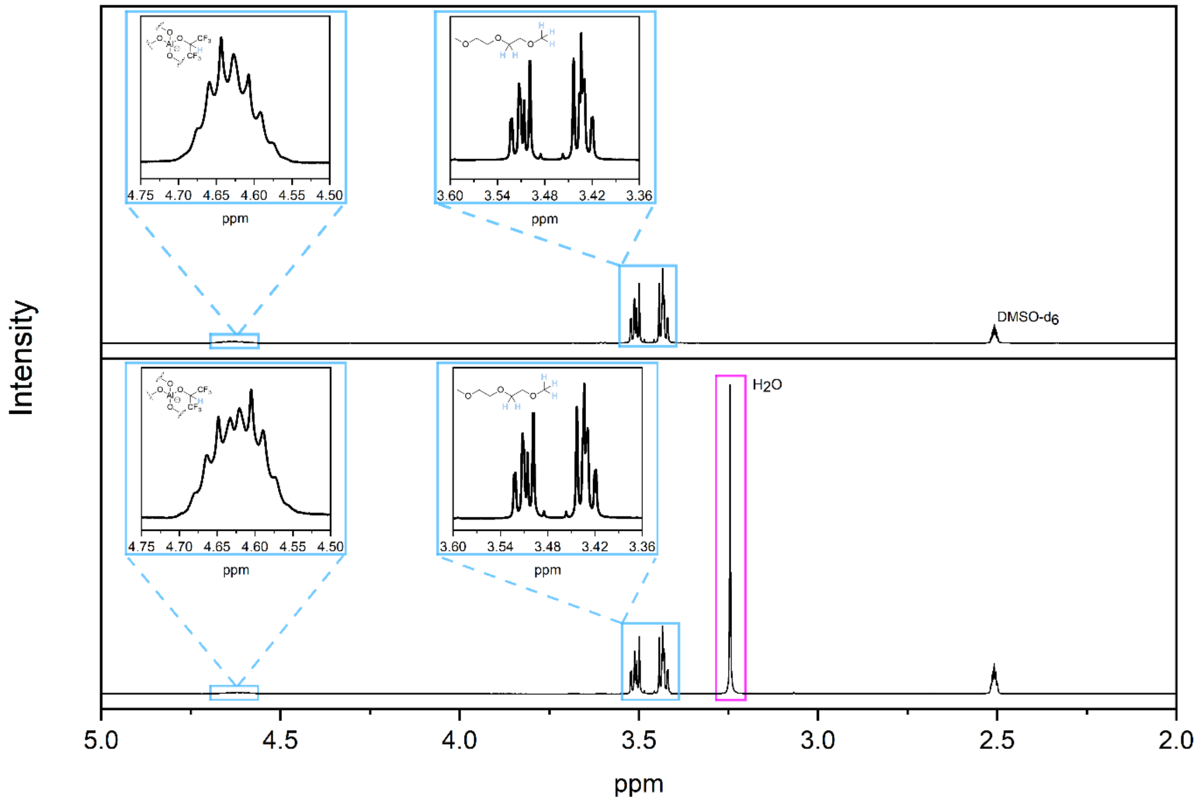

Figure S7: ^1H NMR spectra of 0.4 M MgAlhfip/G2 electrolyte with 0 ppm (above) and 1000 ppm (below) of water. Addition of water does not initiate any anion decomposition.

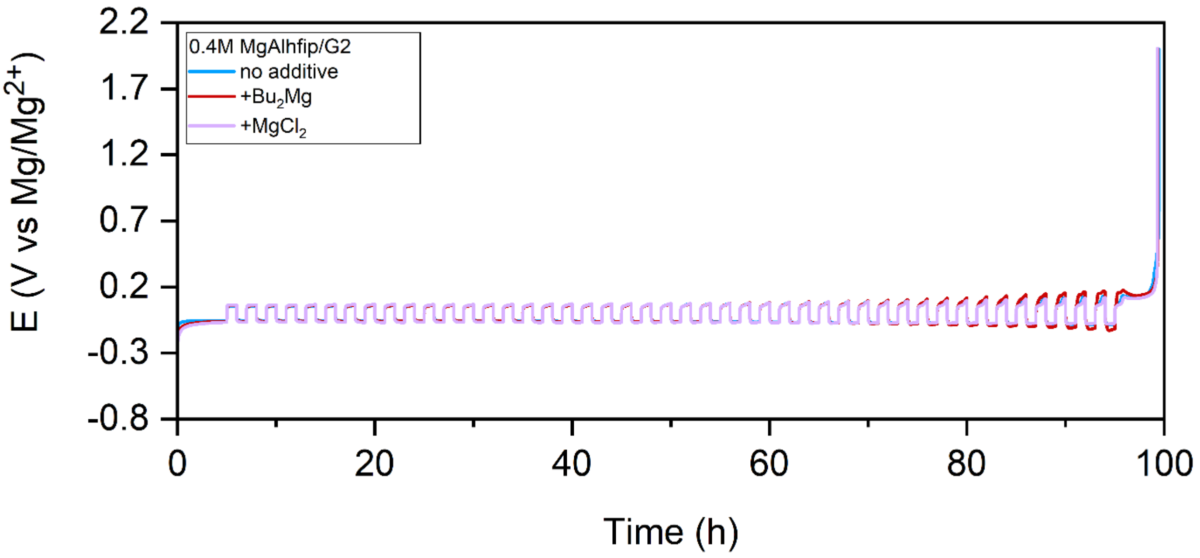

Figure S8: Galvanostatic curves obtained upon macro cycling of Mg electrodes in 0.4 M MgAlhfip/G2 electrolytes without and with 40 mM of additives (Bu_2Mg and $MgCl_2$). Current density 1 mA cm^{-2}, initial 5 h Mg plating followed by regular cycling (1 h Mg stripping and 1 h Mg plating) and final stripping of remaining Mg until overpotential of 2 V.

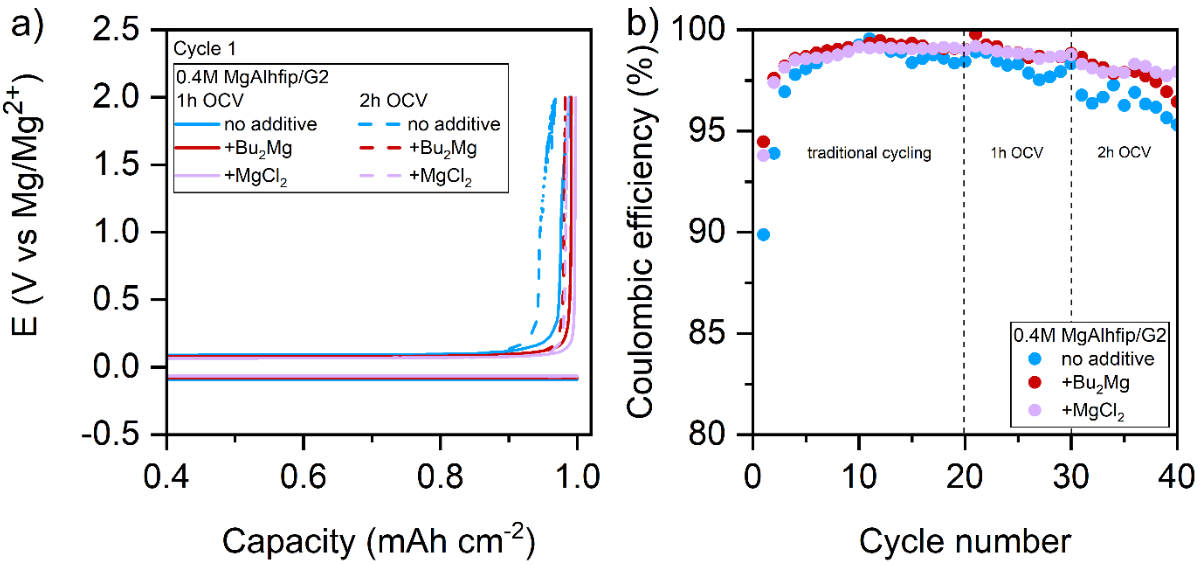

Figure S9: a) Galvanostatic curves related to Mg plating/stripping in the 1st cycle after pauses of 1 (solid) and 2 h (dashed) OCV period in 0.4 M MgAlhfip/G2 electrolytes without and with 40 mM of additives (Bu_2Mg and $MgCl_2$). Current density 1 mA cm^{-2}, 1 h Mg plating followed by stripping until overpotential of 2 V, b) Coulombic efficiency of Mg plating/stripping. Dashed lines are marking the areas of different cycling protocols; left: traditional cycling, middle: cycling with 1 h OCV period after each Mg plating, right: cycling with 2 h OCV period after each Mg plating.
